# New York Pasteur Institute

**Published:** 1890-11

**Authors:** 


### NEW YORK PASTEUR INSTITUTE.

---

Dr. Paul Gibier, director of the New York Pasteur Institute, begs to inform you of the preventative inoculations against hydrophobia performed at this institute since its opening (February 18, 1890.)

To date 610 persons, having been bitten by dogs or cats came to be treated. These patients may be divided in two categories:

1st. For 480 of these persons it was demonstrated that the animals which attacked them were not mad. Consequently the patients were sent back after having had their wounds attended, during the proper length of time, when it was necessary. *400 patients of this series were consulted or treated gratis.*

2d. In 130 cases the anti-hydrophobic treatment was applied, hydrophobia having been demonstrated by veterinary examination of the animals which inflicted bites or by the inoculation in the laboratory, and in many cases by the death of some other persons or animals bitten by the same dogs. All these persons are, to-day, enjoying good health. *In eighty cases the patients received the treatment free of charge.*

The persons treated were: 64 from New York; 12 from New Jersey; 12 from Massachusetts; 8 from Connecticut; 9 from Illinois; 3 from Missouri; 3 from North Carolina; 3 from Pennsylvania; 2 from New Hampshire; 2 from Georgia; 2 from Texas; 1 from Maryland; 1 from Maine; 1 from Kentucky; 1 from Ohio; 1 from Arizona; 1 from Iowa; 1 from Nebraska; 1 from Arkansas; 1 from Louisiana; 1 from Ontario, Can.

